# Features and Risk Factors of Nonfatal Injury among the Rural Children: A Survey of Seven Schools in a Mountain Area in Southwest China

**DOI:** 10.1371/journal.pone.0102099

**Published:** 2014-07-10

**Authors:** Xiu-Quan Shi, Yong-Hong Qi, Dan Shi, Cheng Yan, Junxin Shi, Bo-Ling Cao, Dan Liu, Li-Rong Luo, Hai-Yan Wang

**Affiliations:** 1 Epidemiology and Health Statistics, School of Public Health, Zunyi Medical College, Zunyi, China; 2 Center for Disease Control and Prevention of Zunyi City, Zunyi, China; 3 Center for Injury Research and Policy, Research Institute at Nationwide Children's Hospital, The Ohio State University College of Medicine, Columbus, Ohio, United States of America; 4 Affiliated Hospital of Zunyi Medical College, Zunyi, China; Brighton and Sussex Medical School, United Kingdom

## Abstract

**Objective:**

We aimed to investigate the patterns and risk factors of nonfatal injuries among rural mountain-area children in southwest China.

**Methods:**

A stratified sampling method was used to recruit rural children aged 8 to 17 years (mainly 9–14 years) from 7 schools. Self-reported injuries during the past 12 months and relevant concerns were collected from June to December 2012 by using a structured questionnaire in a class interview.

**Results:**

The mean age of the 2,854 children was 12.2±1.5 years. The probability of annual injury was 16.7% (95% confidence interval [95% CI] 15.3–18.1%), with slightly higher injury risk for boys than girls (17.7% vs. 16.0%; *P*>0.05). The top 3 causes of injuries were falls (37.3%), animal-related incidents (20.6%), and burns (14.9%). The main injury risk factors included being involved in a violent episode (odds ratio [OR] 1.34, 95% CI 1.08–1.66, *P* = 0.007), maltreatment by parents or guardians (1.42, 1.17–1.72, *P*<0.001), and being from a single-child family (1.30, 1.10–1.66, *P* = 0.039). Older age was a protective factor (0.81, 0.76–0.87, *P*<0.001).

**Conclusions:**

The incidence of nonfatal injury among rural children was high, and falls were the leading cause. Younger children and boys from poor-care and poor-living environments were at increased risk of injury, which requires urgent attention. Injury prevention programs targeting these issues are needed in this mountain area and similar rural regions of China.

## Introduction

Injury is a worldwide public health problem that affects all ages of the population. The World Health Organization and United Nations International Children's Fund indicated that worldwide, almost half of the pediatric population has had at least one experience with injury. Moreover, injuries among children and adolescents are the leading cause of premature death, disability, medical costs and lost productivity [1], [2]. Globally, nonfatal injuries are a significant cause of childhood morbidity; most injuries occur in low- and middle-income countries [2], [3].

As the most populous developing country, China has an annual incidence of injuries for all ages of between 16.1% and 21.9% based on data from the China Ministry of Health [4], [5], and 700,000 to 750,000 people die due to injuries each year. Although fatal injuries have been well examined because of severe outcomes, nonfatal injuries are more common than fatal injuries, which has an enormous impact on children and their families. The rates of the main portion of nonfatal injury, unintentional injury, range from 11.3% to 13.9%, depending on the region, with the injury rates in rural areas slightly higher than those in urban areas [6]. In a word, injuries have led to a heavy burden of disease and have become a major public health problem in many developing countries including China [7]–[9].

Children in rural areas of China are more vulnerable to injury than are those in urban areas and tend to have much different injury patterns as compared with urban children. Rural children are exposed to open water, animals, mountainous regions, and other natural hazards [9], [10]. Moreover, children living in rural areas are likely be poor and have difficulties accessing quality and affordable health care and services; lack of adequate supervision by parents is often an important factor of injury.

In the past several decades, many studies have investigated injuries among rural children and adolescents. One study, conducted in rural central China, found that such children had significantly higher rates of injuries because of lack of supervision; however, the authors mainly compared “left-behind” children (children living with a single parent or their extended family members while their parents seek employment in cities far from their home town) and those living with both parents [11]. Another study in Guangxi province in rural southern China reported that drowning was the primary cause of death in children aged 0 to 14; however, the authors only reported fatal injuries [12].

Some risk factors of nonfatal injuries may be unique to children in rural areas [9], [10]; however, little work has focused on nonfatal pediatric injuries in rural China, especially in western mountain areas, with low-income economies. Whether the injury incidence, injury features and risk factors in this rural mountain area are similar as urban areas or not remain unclear.

In this study, we aimed to test whether rural children of mountain areas may have different injury epidemiological characteristics and risk factors than children living in other areas. We wanted to gather evidence identifying the potential injury risks among such children to highlight our work in developing effective injury prevention programs in similar areas.

## Methods

### Survey design and sampling

This research was conducted in Zunyi city, Guizhou province, southwest China, with jurisdiction over 3 districts and 12 counties in 2012. The area covers 11,882 square miles and is a typical mountain-rich area. It has a population of approximately 7.5 million; about 84.3% were farmers with a mostly agricultural economy in the 2010 census. The area includes about 2 million children under age 18 (27% of the total population).

We used a cross-sectional study design with a multistage cluster sampling method. First, we randomly sampled 3 counties in Zunyi city – Huichuan, Zheng'an, and Meitan – then randomly selected 7 schools (including 3 middle schools and 4 primary schools) from 5 towns in the 3 counties. The schools represented grades 4 through 7. All students aged 8 to 17 years (mainly 9–14 years) were asked to participate if they were in the school during the survey period from June to December 2012.

### Definition of injury

In our survey, we defined an injury if it met one of the following criteria: (1) required medical attention [13] or (2) activities were restricted and rest was required for at least half a day [14] during the 12 months before the interview. In the first criterion, medical services were often offered by the school or village doctors in clinics or hospitals. In fact, the second criterion overlapped with the first criterion (requiring medical attention); we only used it as a supplement. We excluded some very slight injuries requiring no more than half a day's rest using the second criterion.

### Sample size estimation

Considering a possible difference in injury incidence between boys and girls, we divided children into 2 strata and determined the sample size for injury incidence of one stratum by a formula [15]: N = Zα^2^×p×q/(d^2^); where Z is Z-value, α (alpha) is probability of type I error, p×q is the maximum possible estimate of variance, and d (delta) is the permissible error. Considering missing values or non-response, we added 20% to the estimated sample, for a required total sample size of 2,561. We also used an estimation method [16], [17] for sample size for risk factors and estimated a required sample of at least 2,364.

### Data collection and quality control

Data were collected at each school by use of a standard questionnaire. Trained interviewers entered each classroom, explained the purpose of the survey, and obtained informed consent from each student. Questionnaires asked about (1) demographics, including age, gender, grade, and personality (e.g., introverted, extroverted). Considering the assumption of a possible difference in injury between urban and rural children, we paid close attention to the (2) family and school condition, including children from a single-child or multiple-children family, and family status (see below), living surroundings (living near or far away rivers or lakes), seldom or often praised by teachers, parents or guardians; and (3) potential risk factors, including behavioral factors (such as riding bikes, swimming in rivers and/or lakes), violent experiences (violence in this study was defined as any behavior intended to harm children psychologically or physically while in school or near home [<1000 meters]), or maltreatment by parents or guardians. We classified family status into 4 subgroups: living with parents, father or mother only, or someone other than parents (last 3 types classified as left-behind children [11]).

The outcome in our survey was whether a child had an injury during the previous 12 months. For each reported injury, children were asked about the cause of injury, type of injury, injury place, and body part injured, activity at the time of injury, whether and how any medical treatments were sought, and the outcome of injuries. Teachers were present in the classrooms during the interview to help children understand the questions if necessary but did not help them answer the questions.

All interviewers were staff or post-graduate students majoring in public health or clinical medicine. They were trained and simulated interviews with each other several times before performing the actual interviews. The principal researchers visited the field to supervise the survey activities. The principal researchers and all interviewers reviewed the completed questionnaires for accuracy and completeness during the survey.

### Ethics statement

A written informed consent was obtained from each student and parents or legal guardian(s). The research protocol and questionnaires were approved by the 7 targeted schools and the Institutional Review Board of Zunyi Medical College.

### Statistical analysis

A database was constructed by use of EpiData 3.1 (http://www.epidata.dk). Data were double-entered to reduce errors. Statistical analyses involved use of SPSS v18.0 (SPSS Inc., Chicago, IL, USA). Data were described with number (percentage) and mean ± SD. The trend of nonfatal injuries by grade was tested by the Cochran-Armitage trend test. Injury risk factors were explored by univariate and multiple logistic regression models. A stepwise method was used for variable selection in the multi-variable model. Two-sided *P*<0.05 was considered statistically significant.

## Results

### General characteristics of injuries

Our study strategy yielded a sample of 2,854 children (response rate 99.6%) in 7 schools (mean age 12.2±1.5 years) to complete the baseline survey. The sample approximately matched the distribution of school-aged children in Zunyi city. In all, 477 students reported 1 or more injuries in the previous 12 months, with an annual incidence of nonfatal injuries of 16.7% (95% confidence interval [95% CI] 15.3–18.1%; Table 1): 320 students reported 1 injury, 88 reported 2 injuries, and 69 reported as least 3 injuries. Overall, 94.5% of injuries were unintentional (incidence 15.8%, 95% CI 14.5–17.1%); the intentional injuries (including violence-related and self-inflicted injuries) accounted for 0.9%.

**Table 1:** Injury incidence among school-aged rural children by school grade, family status and sex in rural southwest China.

Variables	Male		Female		Total injury	P-value*
	Injuries, No. /	Injury	Injuries, No. /	Injury	incidence (%)	
	total students	incidence (%)	total students	incidence (%)		
**Grade**						<0.001^a^
4	53/180	29.4	50/167	29.9	29.7	>0.05^c^
5	72/373	19.3	67/347	19.3	19.3	>0.05^c^
6	48/271	17.7	48/249	19.3	18.5	>0.05^c^
7	80/616	13.1	59/651	9.1	11.0	= 0.027^c^
**Guardian(s)**						= 0.186^b^
Both parents	103/577	17.9	97/592	16.4	17.1	>0.05^c^
Father-only	24/120	20.0	20/113	17.7	18.9	>0.05^c^
Mother-only	39/274	14.2	31/245	12.7	13.5	>0.05^c^
Other than parents	87/469	18.6	76/464	16.4	17.1	>0.05^c^
**Total**	253/1440	17.7	224/1414	16.0	16.7	= 0.216^c^

*Different letters indicate significant difference by grade^a^, guardian situation^b^, gender^c^.

The overall incidence was slightly higher for boys than girls (17.7% vs 16.0%, *P*>0.05). For grades 4, 5 and 6 (age 11.1±1.4 years old, primary school stage), the incidence of injuries did not differ between boys and girls. However, for children in grade 7, the incidence was higher for boys than girls (mean age 13.6±1.3 years old, middle school stage) (13.1% vs 9.1%, *P* = 0.027). For both boys and girls, the incidence of nonfatal injuries showed a trend to decrease with increasing age or grade (Cochran-Armitage trend test, *P*<0.001; Table 1).

Almost two thirds of children (n = 1,831; 64.2%) reported that their immediate family member(s), mostly fathers, were working outside their hometown (in other cities and even other provinces). For 45.3% of children, the parent would come back home to see their children every 6 months, and for 39.9%, the parent could not even visit their family once during the year. So children were mostly taken care of by a single parent or other guardians such as grandparents and older brothers or sisters. The mean age of the principal guardian was 41.0±11.7 years (range 10–84 years).

Overall, 1,685 children were classified as left-behind children: 55.4% (933 children) had both parents absent, 13.8% (233 children) were living with their father only and 30.8% (519 children) their mother only (Table 1). The annual injury incidence did not differ by family status except that the incidence was higher, with marginal significance, for children living with their father only than mother only (18.9% vs 13.5%, *P* = 0.056, Table 1). Moreover, 491 students (17.2%) were from a single-child family, and most (98.5%) needed to do some housework.

### The nature of injury (cause/mechanism and location)

The top 5 causes of injuries were falls (37.3% of all reported injuries), animal-related injuries (20.6%), burns (14.9%), sharp instruments (11.1%) and traffic accidents (6.9%) (Table 2). Falls was the most common external cause of injuries, and the incidence was also the highest (6.2%).

**Table 2:** The nature of injuries among school-aged rural children in southwest China.

Injury cause (mechanism)			Location of injury				Activities when injured			Injured part of body		
Top 5 injuries	No.	%	Top 5 locations	No.	%		Top 5 activities	No.	%	Top 3 parts	No.	%
Falls	178	37.3	Home	207	43.8		Playing	161	34.5	Hand/arms	183	38.8
Animal-related	98	20.6	School	77	16.3		Walking	103	22.1	Feet/leg	174	36.9
Burns	71	14.9	Field/outdoors	58	12.3		Housework	69	14.7	Head	60	12.7
Sharp instrument	53	11.1	Road/street	46	9.7		Riding bicycle	52	11.1	-	-	-
Traffic accident	33	6.9	Public play place	30	6.3		Sport	40	8.6	-	-	-
Other	44	9.2	Other	55	11.6		Other	42	9.0	Other	55	11.6
Total	477	100.0	Total*	473	100.0		Total*	467	100.0	Total*	472	100.0

*Sample size <477 due to missing values.

As we might expect, children's injuries (mainly unintentional injuries) occurred in various places, including at home (43.8%), at school (16.3%), outdoors such as in farm fields (12.3%), on the roads to school (9.7%), and in public recreational places (6.3%) (Table 2). So, the most frequent locations for injuries and the places with greatest risk were places children spent most of their time. For boys, the injuries were most likely to occur in farm fields and outdoors and for girls at home, but the gender differences in locations of the injury was not significant (*P*>0.05).

Because we focused on the activities children were performing when the injury occurred, the top 5 most-common activities were playing (34.5%), walking (22.1%), doing housework (14.7%), riding bicycles (11.1%), and doing sports and other activities (17.6%). The leading injured parts of body were hands or arms, then feet or legs, heads, and other areas. Injuries to limbs accounted for more than three-quarters of the total injuries, and head, trunk, and other parts accounted for less than one-quarter.

### Risk factors of injury

On univariate analysis, we found 3 potential risk factors of injury in addition to 5 selected factors in the multi-variable model (Table 3): being from a single- than multiple-child family (20.0% vs 16.2%, odds ratio [OR] 1.30, 95% CI 1.10–1.66, *P* = 0.039), having an extroverted than introverted personality (1.13, 1.01–1.28, *P* = 0.044) and living near rather than far from rivers and/or lakes (1.33, 1.06–1.71, *P* = 0.025).

**Table 3:** Risk factors of injury among school-aged rural children.

Factors#	Univariate model	Multiple model	Wald χ^2^	P-value
	(Crude OR and 95%CI)	(Adjusted OR and 95%CI)		
Age (per 1 year older)	0.79 (0.74–0.85)	0.81 (0.76–0.87)	35.57	<0.001
Maltreatment by parents or guardians (yes vs never)	1.57 (1.31–1.89)	1.42 (1.17–1.72)	12.92	<0.001
Violence in surroundings	1.47 (1.23–1.75)	1.35 (1.12–1.63)	9.77	0.002
Being involved in violent episode	1.59 (1.30–1.94)	1.34 (1.08–1.66)	7.27	0.007
Praise from teachers, parents or guardians	0. 69 (0.50–0.95)	0.68 (0.49–0.94)	5.39	0.020
(seldom vs often)				
Living by rivers or lakes (near vs far)	1.33 (1.06–1.71)	___	5.02	0.025
Single-child vs multiple-children family	1.30 (1.10–1.66)	___	4.28	0.039
Personality (extroverted vs introverted)	1.13 (1.01–1.28)	___	4.06	0.044

# The latter factor is the reference.

OR, odds ratio; 95% CI, 95% confidence interval.

Results from multivariate logistic regression analyses revealed 3 risk factors associated with injury incidence, including experiencing violence in the surroundings (OR = 1.35, 95% CI 1.12–1.63, *P* = 0.002); being maltreated by parents or guardians (1.42, 1.17–1.72, *P*<0.001) and being involved in a violent episode (1.34, 1.08–1.66, *P* = 0.007). Protective factors were older age (0.81, 0.76–0.87, *P*<0.001) and receiving little praise from teachers and/or guardians (0.68, 0.49–0.94, *P* = 0.020).

## Discussion

Globally, nonfatal injuries are a significant cause of childhood morbidity, most injuries occurring in low- and middle-income countries [2], [3]. Our data from Zunyi, a predominantly agricultural mountain-rich region of rural southwest China, is valuable for researchers and the public to understand the characteristics of nonfatal pediatric injury in rural China. Our finding of 16.7% incidence of nonfatal pediatric injury in Zunyi is higher than in many countries. For example, in the United States, a prevalence of 10.2% was reported [18]. In the United Kingdom, the mean annual injury rate was 14.4% [19]. In developing countries such as Pakistan (economically comparable to China), the incidence was 12.1% for children in rural areas [20]. Our finding is also higher than in some other regions in China such as Shandong, a coastal province about half rural and half urban in eastern China, with an incidence of 6.8% [14].

The higher incidence in rural southwest China than in other regions may be due to the differences in rural and urban lifestyle; environmental risks (poorly maintained roads and sidewalks, exposure to animals); cultural practices (parents are busy with agricultural tasks and do not have enough time to supervise children); and poor access to medical care. Our findings may differ from those in other areas because although the definitions of injury were similar among studies (needing medical attention), some surveys focused on unintentional injury [9], [10] and others on total injuries, even including fatal injuries [8]; some included serious injuries that must be recorded by hospitals [19]. Our study focused on all nonfatal injuries, which were more numerous than in the Ma et al. study [14] despite a similar injury definition. After excluding 5.5% (26 children) with intentional injuries in our survey, 94.5% of children all experienced unintentional injuries. The injury classification was almost the same as in another Chinese study in the eastern area (Li et al. study [9]), but our unintentional injury incidence was slightly higher (15.8% vs 15.6%).

Our finding that falls are the leading cause of nonfatal injuries is consistent with several findings in China [13], [21], [22] but is different from that in some urban areas, where traffic-related injuries are the leading mechanism of injuries [23]. Southwest China is a mountain-rich area, and only some children in rural Zunyi live near a paved road, and fewer vehicles travel on roads. Walking or bicycling is the most common traffic style. Therefore, children in these areas are prone to falling when they are walking, riding bicycle or playing. In other regions and countries, automobile transport is more common, so road traffic injuries in Zunyi are not as serious as in other regions.

Our risk factors of nonfatal injury may also differ from those in urban areas and mainly included family conditions, supervision, and living surroundings. China has a “one child” policy because of the huge population. Now, most urban families have one child, but in rural areas, the supervision of this policy is not as strict as in urban areas. Some families have more than one child for cultural reasons. Therefore, single- and multiple-child families coexist in rural areas. In many single-child families, parents and especially grandparents may spoil the child and allow the child to do almost everything they want to do. They support the child instead of strictly supervising. The increased injury risk in children from single-child families potentially reflects spoiling the child (children become dependent and have less ability to deal with injury risks).

Receiving little praise from teachers, parents and guardians was a protective factor for injuries. Children may receive less praise possibly because they do less housework and thus are less exposed to injury. Moreover, children with less praise may be more independent and may be able to better deal with the injury risk circumstances. As well, maltreatment by parents or guardians could increase the injury risk, by 42%, on average. Some guardians may consider that physical punishment can teach children a lesson not to make the same mistakes, but physical punishment can sometimes directly result in injury or indirectly affect the child's well-being to prevent injuries. Children who live in single-parent households are believed to receive less parental involvement and supervision than those living in two-parent households [24]. We found injuries slightly higher for children living with only their father than only their mother. These findings differed from those of Shen et al. [11], conducted in a plains area; our study was conducted in a mountainous area, and injury easily occurred for children even with 2 parents at home.

Because we focused on living surroundings, including natural and social surroundings, we found that living near rivers or lakes significantly increased the injury risk. Children, especially boys, like swimming, and being near lakes and rivers put them at risk of drowning. Being far away from violence is believed to be an important social support for children's healthy growth. We found that having violent phenomena in living surroundings could increase the injury risk by 35%; if children were involved in violent episodes, the risk was increased by 34%. Because the association of violence and injuries in children is well demonstrated [25], to lower the incidence of injury in children, we should keep them away from violent activities.

We found that age was a protective factor of pediatric injury (OR = 0.81). The environment in rural China may be less risky for older children. It may be related to cognitive development with increased age and children having a better understanding of complex risk circumstances and therefore reducing their risk behaviors.

In some reports, the ratio of injury incidence for boys was nearly double that for girls [26], [27], but we found no significant difference between boys and girls in injury incidence. In Chinese culture, girls generally need to do more housework than boys, which exposes them to more risk factors such as burns by ovens, hot liquids and foods while they are cooking.

Our study suggests that prevention priority should be given to fall injuries, then animal-related injuries in children in rural China. Younger children and boys from poor-care and poor-living environments may be at higher risk of injury, which requires urgent attention. We should take good care of children, especially rural children from single-child families (good relationships between parents and children are helpful for emotional and cognitive development and could avoid many injuries [28]), and prevent them from living in violent surroundings because of the high risk for injury. Moreover, more efforts should be taken to change the other modifiable injury risk factors we mentioned above.

Our study has some limitations. First, the outcome measure was based on self-reporting, which might underestimate the problem because some minor injuries might be forgotten and not reported. Second, we analyzed the injuries occurring in the previous 12 months. Although 12 months can efficiently avoid the effect of seasonal fluctuation on injury incidence as compared with 3-month recall, the long period may lead to more recall bias because of the long time. Finally, we focused on only school-aged children aged 8 to 17 in rural Zunyi, although we recruited a large sample of children; our results may not be representative of all children in southwest China. Further research and interventions might consider studying injury risk among adolescents and preschool children to extend the findings to all rural children. Moreover, we should further compare our results with urban Zunyi instead of other regions in the future.

## Conclusions

Our study showed that one sixth of rural children in Zunyi in southwest China report an annual injury episode. Our study highlighted the first step of injury prevention in children by attempting to describe the risk. The next step should focus on the injury intervention and health care. Injury intervention programs in China need to target and address these vulnerable children.
